# Occurrence and characterization of biofilm-forming and multidrug-resistant *Shigella* spp. in retail poultry meat and associated environments in Sylhet, Bangladesh

**DOI:** 10.1016/j.psj.2026.107935

**Published:** 2026-10-09

**Authors:** Suchona Aktera, Md. Hasan Alib, Hemayet Hossainc, Md. Shahidur Rahman Chowdhurya, Mohammad Nahian Rahmana, Sumaya Shargin Khana, Afroza Sultana Nitua, Tanvir Ahmada, Md. Mukter Hossaina, Md Bashir Uddina, Md. Mahfujur Rahmana

**Affiliations:** a Department of Medicine, Sylhet Agricultural University, Sylhet, 3100, Bangladesh; b Faculty of Veterinary, Animal and Biomedical Sciences, Sylhet Agricultural University, Sylhet, 3100, Bangladesh; c Department of Anatomy and Histology, Sylhet Agricultural University, Sylhet, 3100, Bangladesh; d Sylhet Agricultural University Sylhet, Bangladesh

**Keywords:** Extended spectrum *β* lactamase, Biofilm formation, Multidrug resistance, Poultry processing environment, *Shigella* spp

## Abstract

*Shigella* spp. are important foodborne pathogens increasingly associated with antimicrobial resistance (AMR) and contamination within retail poultry-handling environments. This study investigated the prevalence, antimicrobial resistance profiles, extended-spectrum β-lactamase (ESBL) determinants, virulence-associated genes, efflux pump genes, and biofilm-forming ability of *Shigella* spp. isolated from retail poultry meat and associated environments in Sylhet, Bangladesh. A total of 300 samples, including cloacal swabs, meat, handler hand swabs, utensil swabs, and wastewater samples, were collected and analyzed using culture-based methods, biochemical tests, and species-specific PCR. We also performed antimicrobial susceptibility testing (AST) and molecular characterization. Overall, 50.0% (150/300) of samples were culture-positive for *Shigella*. Among the 150 culture-positive samples, 144 (96.0%) were confirmed by species-specific PCR, including 98 *Shigella flexneri* and 46 *S. dysenteriae* isolates. When calculated against the total number of samples tested, *Shigella flexneri* and *S. dysenteriae* were present in 32.67% (98/300) and 15.33% (46/300) of samples, respectively, with the highest prevalence observed in cloacal swabs (46.38% and 21.74%, respectively). AST revealed high resistance to trimethoprim-sulfamethoxazole (92.9% and 84.8%), tetracycline (87.7% and 87.0%), azithromycin (86.7% and 76.1%), and levofloxacin (76.5% and 56.5%) among *S. flexneri* and *S. dysenteriae*, respectively. In contrast, cefixime and gentamicin remained the most effective agents, showing susceptibility rates of 82.7% and 80.6% in *S. flexneri* and 67.4% and 58.7% in *S. dysenteriae*. The predominant resistance genes were *tetA* (72.45; 78.26%), *qnrS* (80.61; 89.13%), *sul1* (50.0; 69.57%), and *bla*_TEM_ (38.78; 45.65%), while efflux pump genes *acrAB* (60.20; 80.43%) and *tolC* (77.55; 86.96%) were widely distributed. Biofilm formation was detected in 37.76% and 56.52% of isolates by Congo red agar and in 27.55% and 36.96% by microtiter plate assay for *S. flexneri* and *S. dysenteriae*, respectively. All isolates exhibited MAR indices >0.20, while MDR and possible XDR (P-XDR) phenotypes were detected in 38.78% and 61.22% of *S. flexneri* and 58.70% and 41.30% of *S. dysenteriae* isolates, respectively. These findings highlight the occurrence of antimicrobial-resistant and biofilm-forming *Shigella* spp. within the investigated retail poultry-handling environment and underscore the need for strengthened biosecurity, antimicrobial stewardship, and One Health surveillance strategies.

## Introduction

The poultry industry is one of the fastest-growing agricultural sectors worldwide and plays a critical role in food security, nutritional supply, and economic development, particularly in low- and middle-income countries such as Bangladesh (Raquib et al., 2021; Hashem et al., 2024). However, the expansion and intensification of poultry production have also increased the burden of foodborne bacterial contamination and environmental waste generation, raising significant concerns regarding zoonotic pathogen transmission and food safety (Sharma et al., 2025). Poultry meat may become contaminated at multiple stages, including slaughter, processing, transportation, and retail handling, while poultry-associated environmental sources such as wastewater, utensils, litter, and worker-contact surfaces may serve as reservoirs for bacterial persistence and dissemination (Rouger et al., 2017). These conditions create ideal opportunities for virulent, drug-resistant enteric pathogens to enter the human food supply.

*Shigella* spp. remain a major global cause of bacterial diarrhoea, with an estimated 80–165 million infections annually, disproportionately affecting children under five years of age in low- and middle-income countries (WHO, 2005). The genus comprises four principal species *S. dysenteriae, S. flexneri, S. boydii,* and *S. sonnei*, all of which are facultatively anaerobic, Gram-negative enteric pathogens responsible for shigellosis (Shi et al., 2014). Following invasion of the intestinal epithelium, *Shigella* induces focal mucosal destruction, resulting in micro-ulceration, inflammatory exudation, and the presence of blood and polymorphonuclear leukocytes in stool (Gaurav et al., 2013). Clinically, infection ranges from mild watery diarrhoea to severe bacillary dysentery, with complications such as haemolytic–uraemic syndrome, sepsis, and post-infectious reactive arthritis (Taylor, 2008; Ajene et al., 2013). Among the species, *S. dysenteriae* is particularly associated with severe outbreaks and higher mortality rates (Raja et al., 2011), partly due to its extremely low infectious dose of approximately 10–100 organisms (Matanza and Clements, 2023; Samandari and Okafor, 2025). These traits underscore *Shigella*'s high public-health importance and justify stringent surveillance at the human-animal-environment interface. Poultry meat and processing environments are increasingly recognized as potential vehicles for *Shigella* exposure, despite the genus being classically viewed as human-adapted (Rouger et al., 2017).

The virulence arsenal of *Shigella* is central to its pathogenicity. Chromosomal and plasmid-borne determinants, including invasion plasmid antigen (*ipa*) genes, *virF* (a transcriptional activator), the type III secretion system (*T3SS*) and its effectors, and enterotoxins (*set, sen*), mediate epithelial invasion, intracellular replication, actin-based motility, and immune evasion (Schroeder and Hilbi, 2008; Mattock and Blocker, 2017; Trirocco et al., 2023). Together, these factors enable *Shigella* to destroy colonic epithelium, induce intense inflammation, and cause bloody diarrhea (Mattock and Blocker, 2017). Variations in the presence or expression of these virulence factors directly influence disease severity, transmissibility, and the likelihood of sustained outbreaks (Dorman, 2004; Pakbin et al., 2023). Consequently, virulence profiling of *Shigella* isolates from poultry and environmental sources is imperative for distinguishing between incidental environmental contamination and genuine human pathogens with high clinical threat.

Antimicrobial resistance (AMR) exponentially amplifies the public-health threat posed by *Shigella* (CDC, 2024). The genus exhibits a precipitous global rise in resistance to first-line antibiotics, ampicillin, tetracyclines, trimethoprim–sulfamethoxazole, and escalating non-susceptibility to fluoroquinolones and third-generation cephalosporins (Ahmed et al., 2023). Resistance mechanisms are predominantly plasmid-mediated, facilitated by mobile genetic elements that accelerate horizontal gene transfer within Enterobacteriaceae (Partridge et al., 2018; Baker et al., 2018; Hossain et al., 2026). Invasion of multidrug-resistant (MDR) *Shigella* into the food chain would catastrophically constrain treatment options while elevating healthcare costs, morbidity, and mortality (CDC, 2024; Bengtsson et al., 2022). The poultry sector represents a documented reservoir and amplifier of AMR, wherein high antibiotic utilization for prophylaxis and growth promotion generates selective pressure favoring MDR strains and potentially co-selecting virulence determinants (Farkas et al., 2025). Recent molecular profiling of MDR *Shigella* from chicken farms illuminates the concerning convergence of virulence and resistance within a foodborne context (Jihad and Al-Shattrawi, 2025).

Biofilm formation is a key persistence trait that may enhance *Shigella* survival in food-handling and retail meat environments, potentially complicating sanitation and contamination control (Ellafi et al., 2011; Giaouris et al., 2014). Biofilms protect bacteria from desiccation, disinfectants, and antimicrobial exposure while providing a niche that facilitates horizontal gene transfer (Berlanga and Guerrero, 2016). *Shigella* can develop biofilms under food-relevant environmental conditions such as nutrient limitation, bile exposure, and surface attachment, with biofilm-associated states often showing increased antimicrobial tolerance and maintenance of virulence determinants (Nickerson et al., 2017; Chiang et al., 2021). In poultry processing facilities, characterized by moisture, organic residues, and hygiene challenges, biofilms on equipment and carcass surfaces may contribute to persistent contamination and intermittent dissemination of viable cells into the food chain (Dass and Wang, 2022). Collectively, the convergence of biofilm-mediated persistence with antimicrobial resistance and virulence traits heightens the risk of sustained food-chain contamination and human exposure.

Despite the expanding poultry sector in Bangladesh, data on the occurrence and characterization of *Shigella* in poultry meat and associated environmental sources remain limited, particularly at the regional level. Information regarding virulence-associated traits, antimicrobial susceptibility profiles, and biofilm-forming ability of poultry-associated *Shigella* isolates is still scarce. Therefore, the present study aimed to isolate and identify *Shigella* spp. from poultry meat and poultry-associated environmental sources and to characterize their virulence traits, antibiotic susceptibility patterns, and biofilm-forming ability.

## Materials and methods

### Ethical Statement

The study received ethical approval from the Institutional Ethics Committee of Sylhet Agricultural University, Sylhet 3100, Bangladesh under Animal Use Protocol No. AUP2025006. All experimental procedures were conducted by trained personnel in accordance with the university’s ethical standards and regulatory guidelines. Written consent was taken before collection of hand swab from chicken handlers. Throughout the study, strict measures were implemented to ensure the welfare and humane treatment of all animals involved.

### Study design, location, and period

A cross-sectional study was conducted from September 2025 to November 2025 in retail poultry shops in Sylhet Sadar Upazila, Sylhet District, Bangladesh (Fig. 1). A list of eligible retail poultry shops operating during the study period was prepared with assistance from local livestock/poultry market information and field reconnaissance. Eligible shops were those involved in poultry slaughtering and retail meat handling. Shops were selected using simple random sampling from the identified sampling frame, and samples were subsequently collected from the selected shops according to the predefined sampling scheme.

**Fig. 1:**
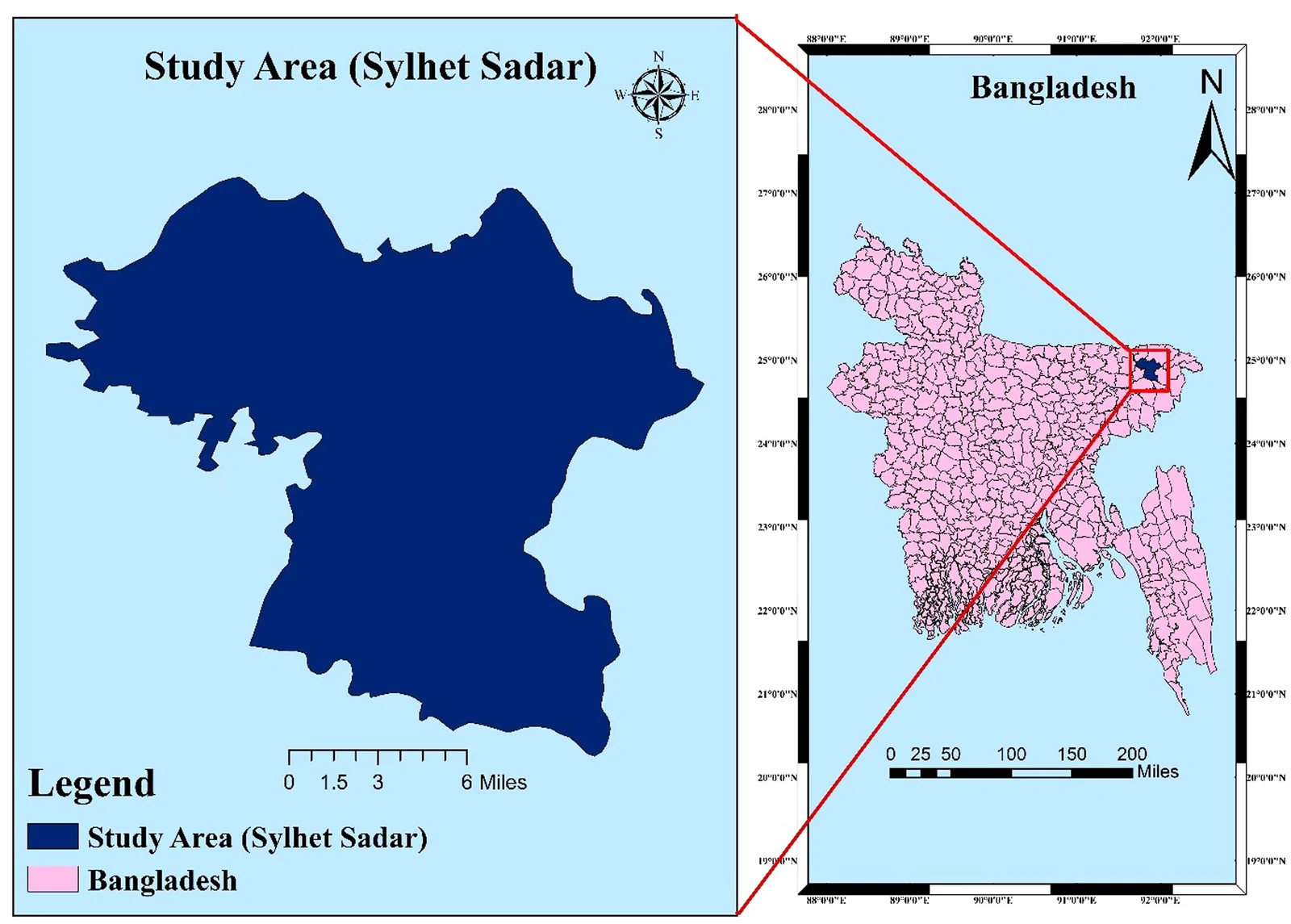
Study area showing the locations where samples were collected. The shapefile was extracted from DIVA-GIS and processed using Geographic Information System (GIS) software to develop the map in ArcMap 10.7 (ArcGIS, ESRI, USA).

### Sample size determination

The required sample size was calculated using Cochran’s (Cochran, 1977) formula:

$$n=\frac{Z^{2}\times P\;\times \;\left(1-P\right)}{D^{2}}$$

Here, Z = 1.96 (95% confidence level)

P = 6.9% = 0.069; The expected prevalence was considered 6.9% as reference article to estimate the minimum number of required samples (Sackey et al., 2001).
D = 5% = 0.05 (precision 5%)

The minimum required sample size was calculated as 98.8 (approximately 99) based on an expected prevalence of 6.9%, a 95% confidence level, and 5% precision. To improve the reliability and statistical robustness of the findings, the sample size was increased to 300.

### Collection and preparation of samples

A total of 300 samples were collected randomly from various sources, including chicken meat and cloacal swab and environmental sources including utensil-associated swabs (Knife, tray, and cage surfaces), wastewater swabs, and hand swabs from chicken handlers, in different butcher shops. Different sample types were collected from the same retail shops when eligible samples were available during field visits. Samples were not systematically linked to specific poultry batches, suppliers, slaughter lots, or source farms; therefore, source-level clustering could not be evaluated. Before sample collection, each sample collecting container is filled with 10 ml of Buffered peptone water (BPW). Then, the designed sample was collected and placed into the corresponding BPW-filled container with appropriate labeled. Samples were aseptically collected by gently swirling a sterilized cotton swab and immediately transferring it into sterilized zip-locked bags, then transported to the department of Medicine by maintaining an appropriate cold chain.

### *Isolation and identification of Shigella* spp.

All environmental and meat swab samples were processed using the same isolation procedures following (Ahmed and Shimamoto, 2014). Following overnight enrichment, a loopful of the enriched broth was aseptically transferred and streaked onto *Salmonella-Shigella* (SS) agar (HiMedia, India) plate for bacterial isolation. Following inoculation, the plates were transferred to an incubator and left for 18-24 hours at 37°C. Colonies that were large, circular, convex, and transparent on SS agar plates were considered to be *Shigella* spp. and culture positives samples were sub-cultured on Xylose Lysine Deoxycholate (XLD) agar, where clear, colorless colonies are observed. To confirm further, stab culture was performed on nutrient agar and incubated. Plates that show motility were discarded because *Shigella* spp. is non-motile. The isolates were sub-cultured several times to obtain pure cultures. Once pure colonies were obtained, they were grown on nutrient agar. After that, a portion from each plate was preserved for storage purpose for further procedures, and another portion was transferred into an eppendorf tube that contain 200 ml of distilled water for genomic DNA extraction.

Gram staining and biochemical assays, including methyl red (MR), motility, citrate utilization, triple sugar iron (TSI), indole, and Voges–Proskauer (VP) tests, were performed on purified presumptive isolates following the relevant standard procedures (Uyttendaele et al., 2001). Presumptive identification was based on colony morphology on selective media, Gram-negative staining, non-motility, and a biochemical profile consistent with *Shigella* spp. (typically MR-positive, citrate-negative, VP-negative, and TSI without H₂S production; indole reactions may vary) (Supplementary Table 1). These findings were considered presumptive rather than definitive species identification. Species-level identification of *S. flexneri* and *S. dysenteriae* was subsequently established using the corresponding species-specific PCR targets, *rfc* and *rfpB*, respectively.

A large, clear, colorless, and circular colony on SS agar, XLD agar plate was suspected as shigella. Representative colony morphology on SS and XLD agar and biochemical tests of *Shigella* isolates are presented in Supplementary Fig. 1.

### Genomic DNA extraction

Genomic DNA of *Shigella* spp. was extracted purified colonies recovered from culture-positive samples using the standard boiling method, as previously described in earlier studies, with slight modifications (Aldous et al., 2005). In brief, two colonies from overnight culture were suspended in 1 ml of distilled water and boiled in a water bath at 100°C for 10 min. The lysate was subsequently centrifuged at 10,000 rpm for 5 min, and the clear supernatant was collected and used as the DNA template for PCR amplification. The concentration and purity of the extracted DNA were determined spectrophotometrically using a NanoDrop 2000c instrument (Thermo Fisher Scientific, Waltham, MA, USA). Thus, species-specific PCR was performed only on isolates recovered from culture-positive samples, rather than directly on the original samples.

### *Molecular detection of Shigella* spp*. by multiplex PCR*

Species-specific primers were employed for the amplification of the *rfc*, and *rfpB* genes of *S. flexneri*, and *S. dysenteriae* respectively (Table 1). Each PCR reaction was carried out in a final volume of 25 µL, consisting of 12.5 µL of 2 × master mix, 3 µL of primer mix, 5 µL of DNA template, and 4.5 µL of nuclease-free water (HPLC grade). Multiplex PCR was performed in a thermal cycler (DLAB Scientific Inc., Alhambra, CA, USA) under the following cycling conditions: initial denaturation at 94°C for 3 min; 35 cycles of denaturation at 94°C for 30 s, annealing at 60°C for 30 s, and extension at 72°C for 30 s; followed by an additional annealing step at 60°C for 30 s and a final extension at 72°C for 3 min. Representative agarose gel electrophoresis images showing amplification of species-specific target genes for *S. flexneri* and *S. dysenteriae* are provided in Supplementary Fig. 2. A confirmed positive isolate from the laboratory repository was used as the positive control, while the negative control contained all PCR components except the DNA template, i.e., PCR master mix, primers, and nuclease-free water.

**Table 1:** List of primers along with amplicon sizes, annealing temperature used in this study.

**Target**	**Gene**	**Primer sequences (5′−3′)**	**Amplicon Sizes (bp)**	**Tm**°**C**	**References**
*S. flexneri*	*rfc*	F: TTTATGGCTTCTTTGTCG GC R: CTGCGTGATCCGACCATG	537	60	(Ojha et al., 2013)
*S. dysenteriae*	*rfpB*	F: TCTCAATAATAGGGAACACAG C R: CATAAATCACCAGCAAGGTT	211	60	
Antiboiotic-resistant genes	*tetA*	F: GGCGGTCTTCTTCATCATGC R: CGGCAGGCAGAGCAAGTAGA	502	63	(Siddiky et al., 2021)
	*sul1*	F: CGGCATCGTCAACATAACCT R: TGTGCGGATGAAGTCAGCTC	433	66	
	*strA*	F: ATGGTGGACCCTAAAACTCT R: CGTCTAGGATCGAGACAAAG	893	63	
	*aac(3)-V*	F: TGCTGGTCCACAGCTCCTTC R: CGGATGCAGGAAGATCAA	653	63	
	*qnrS*	F: ACGACATTCGTCAACTGCAA R: TAAATTGGCACCCTGTAGGC	417	55	(Shetty et al., 2025)
ESBL-Producing genes	*bla*_TEM_	F: CATTTCCGTGTCGCCCTTATT R: CGTTCATCCATAGTTGCCTGAC	800	55	(Rahman et al., 2024)
	*bla*_SHV_	F: AGCCGCTTGAGCAAATTAAAC R: ATCCCGCAGATAAATCACCAC	713	55	
	*bla*_OXA_	F: GCACCAGATTCAACTTTCAAG R: ACCCCAAGTTTCCTGTAAGTG	564	55	
	*bla*_CTX-M-group1_	F: TTAGGAARTGTGCCGCTGCGTA R: CGATATCGTTGGTGGTGACCAT	688	55	
	*bla*_CTX-M-group2_	F: CGTTAACGGCACGATGAC R: GATATCGTTGGTGGTGACCAT	404	55	
	*bla*_CTX-M-group9_	F: TCAAGCCTGCCGATGTCGGT R: TGATTTCGCCGTGTAAG	561	55	
	*MultiCase*_ACC_	F: CACCTCCAGCGAATCTGTTTAC R-GTTAGCCAGCATCACGATCC	346	55	
	*MultiCase*_DHA_	F: GCAACAACGACAATCCATCCT R-GGGATAGGCGTAACTCTCCCAA	895	55	
	*MultiCase*_MOX_	F: TGATGGCAACACGGAATTTC R: GCTTTGACTCTTTCGGTATTCG	997	55	
Virulent genes	*ipaB*	F: CAAGCCCTGAATCCGATCAT R: TGTTTACCAACAGTGGCTAC	194	53	(Karimi-Yazdi et al., 2020)
	*ipaC*	F: GGGATGCTTCCTCTGCTTGT R: CCTAGCAGCCCTATCATCAAG	518	55	
	*ipaD*	F: AAGAAGCCGAGCTTGATGGA R: CCTCGCCATTTCCACCTAGA	450	55	
	*Sen (ShET-2)*	F: GCGATGCGTATGGGATTGAC R: CTCCCATTCCTTCCGCAGTT	244	57	
	*virA*	F: GCCTGCACCAGAAGTTATTG R: AGTGTCGTTTTTAGGGACAACT	550	54	
Efflux pump genes	*acrAB*	F: ATCAGCGGCCGGATTGGTAAA R: CGGGTTCGGGAAAATAGCGCG	312	53	(Wasfi et al., 2016)
	*tolC*	F: ATCAGCAACCCCGATCTGCGT R: CCGGTGACTTGACGCAGTCCT	527	51	

### Antimicrobial susceptibility testing

Antimicrobial susceptibility testing (AST) was conducted utilizing the Kirby–Bauer disk diffusion technique on Mueller–Hinton agar (MHA) plates (HiMedia Laboratories Pvt. Ltd., Mumbai, India) in accordance with the Clinical and Laboratory Standards Institute (CLSI) guidelines M100 breakpoints for Enterobacterale (CLSI, 2023) . Ten antimicrobial agents representing nine distinct classes were employed (Supplementary Table 2). A representative Kirby–Bauer disk diffusion assay illustrating antimicrobial susceptibility testing of Shigella isolates is shown in Supplementary Fig. 3. *E. coli* ATCC 25922 was used as the quality-control reference strain, and all antimicrobial susceptibility tests were performed in triplicate according to the CLSI, 2023 guidelines for accuracy.

### Phenotypic detection of ESBL by double disk synergy test

Extended-spectrum β-lactamase (ESBL) production was detected using the double-disk synergy test (DDST) following (Liza et al., 2024). Briefly, a 0.5 McFarland bacterial suspension was inoculated onto Mueller–Hinton agar plates. An amoxicillin-clavulanic acid (30 μg) disk was placed at the center, while ceftriaxone (30 μg) and cefotaxime (30 μg) disks were positioned 20 mm (center-to-center) apart. Following incubation at 37°C for 24 h, isolates exhibiting an enhanced inhibition zone (“keyhole effect”) between the cephalosporin and amoxicillin-clavulanic acid disks were considered ESBL producers.

### Molecular detection of resistance genes and **β-lactams** gene producing Shigella spp.

Molecular detection of antibiotic resistance genes (ARGs) and β-lactam genes were performed using PCR techniques. Monoplex PCR assays were conducted to detect the *tetA, qnrS, strA, aac(3)-iv,* and *sul1* genes, which confer resistance to tetracycline, ciprofloxacin, streptomycin, gentamicin, and sulfonamides, respectively (Table 1). For the detection of β-lactam genes, multiplex PCR was employed simultaneously to amplify *bla*_TEM_, *bla*_SHV_, *bla*_OXA_, *bla*_CTX-M-group 1_, *bla*_CTX-M-group 2_, *bla*_CTX-M-group 9_, *MultiCase*_ACC_, *MultiCase*_MOX_, and *MultiCase*_DHA_ genes (Table 1; Supplementary Fig. 4 and 5). The negative controls contained all PCR components, including the PCR master mix, primers, and nuclease-free water, except for the DNA template. No positive control was included for the detection of efflux pump genes.

### Molecular detection of virulent genes

The virulence genes *ipaC* and *ipaD* were simultaneously amplified using multiplex PCR with two specific primer pairs. In contrast, the *ipaB, sen (ShET-2)*, and *virA* genes were individually detected through monoplex PCR.

### Molecular detection of efflux pump genes

The efflux pump genes a*crAB* and *tolC* were detected using individual mono-plex PCR assays (Table 5). Each reaction was prepared in a total volume of 25 µL, comprising 5 µL of genomic DNA template extracted from the bacterial isolates, 12.5 µL of 2 × PCR master mix (Add Bio Inc., Daejeon, Republic of Korea), 1 µL of forward primer and 1 µL of reverse primer (10 pmol/µL each), and 5.5 µL of nuclease-free water (Supplementary Fig. 6). The negative controls contained all PCR components, including the PCR master mix, primers, and nuclease-free water, except for the DNA template. No positive control was included for the detection of efflux pump genes.

### Agarose gel electrophoresis

The all-amplified products were resolved by gel electrophoresis on 1.5–1.8% low-melting-point agarose gels. Distinct bands of specific primers (Table 1) were obtained, with a 100 bp DNA ladder serving as the molecular size marker. The target genes were visualized under UV illumination to confirm the presence and size of the amplicons.

### Qualitative and quantitative detection of biofilm formation

#### Congo-red Agar (CRA) Method

Biofilm formation was evaluated using the Congo Red Agar (CRA) method (Freeman et al., 1989). BHI agar supplemented with 0.8 g/L Congo red and 36 g/L sucrose (HiMedia, Mumbai, India) was used. Isolates were streaked onto CRA plates and incubated aerobically at 37 °C for 24–48 h. Colonies that were crystalline, dry, and black were considered strong biofilm producers, while smooth or red colonies were classified as weak or non-producers (Supplementary Fig. 7).

#### Crystal violet micro-titer plate (CVMP) assay

The biofilm-forming capacity of bacterial isolates was assessed using the Crystal Violet Micro-sstiter Plate (CVMP) assay (Kouidhi et al., 2010). Overnight bacterial cultures were adjusted to 0.5 McFarland standard and diluted 1:100 in Tryptic Soy Broth (TSB) supplemented with 1% glucose to promote biofilm formation. A 96-well flat-bottom microtiter plate was inoculated with 200 µL of the diluted suspension per well. Plates were incubated statically at 37 °C for 24 h. After incubation, non-adherent cells were removed by washing wells three times with phosphate-buffered saline (PBS, pH 7.2). Adhered biofilms were stained with 0.1% crystal violet for 15 min at room temperature, excess stain was washed off with sterile distilled water, and the bound dye was solubilized using 95% ethanol. Biofilm formation was quantified by measuring absorbance at 570 nm (OD570) using a microplate reader. Isolates were classified as non-biofilm (OD ≤ 0.2), weak (0.2 < OD ≤ 0.4), moderate (0.4 < OD ≤ 0.6), or strong (OD > 0.6) biofilm producers (Chowdhury et al., 2025). The set of isolates were tested in triplicate, with a known biofilm-producing isolate from the laboratory repository used as a positive control and sterile TSB supplemented with 1% glucose used as a negative control.

#### Antibiogram profiling

The multiple antibiotic resistance (MAR) index was calculated as the ratio of the number of antibiotics to which an isolate was resistant to the total number of antibiotics tested. MAR values ranged from 0 to 1, with values ≥0.20 indicating exposure to high-risk sources of antimicrobial contamination. Multidrug resistance (MDR) was defined as non-susceptibility to at least one agent in three or more antimicrobial classes, whereas extensively drug-resistant (XDR) isolates were defined as nonsusceptibility to at least one agent in all but two or fewer antimicrobial classes (Magiorakos et al., 2012).

#### Statistical analysis

Statistical analyses were performed using R software (version 4.3.1). Prevalence rates were calculated using standard epidemiological formulas and expressed as frequencies and percentages. The distribution of antimicrobial resistance genes, ESBL genes, efflux pump genes, and virulence genes was compared using Pearson’s Chi-square test of independence, with Fisher’s exact test applied where expected cell counts were <5. Genes exhibiting monomorphic distribution (i.e., zero prevalence in both groups) were excluded from comparative analyses due to lack of variability. For gene-by-gene comparisons, odds ratios (ORs) with 95% confidence intervals (CIs) were calculated as measures of effect size, and P-values were adjusted for multiple comparisons using the Benjamini–Hochberg false discovery rate (FDR) procedure. Because multiple sample types could originate from the same retail poultry shop, potential within-shop dependence was acknowledged; however, source-level clustering could not be formally assessed because samples were not systematically linked to specific poultry batches, suppliers, slaughter lots, or source farms. For binary variables (gene presence/absence and resistant/susceptible phenotypes), pairwise associations were assessed using the phi (φ) coefficient and Fisher’s exact test. Analyses were performed separately by species, with *P*-values adjusted using the Benjamini–Hochberg false discovery rate (FDR) procedure. Two-sided *P* < 0.05 was considered nominally significant, while FDR-adjusted *q* < 0.05 indicated statistical significance. Associations significant at the nominal level but not after FDR correction were considered exploratory.

## Results

### *Prevalence and distribution of Shigella* spp*. across different sample types*

A total of 300 samples (cloacal swabs, handlers, meat, utensils, and wastewater) were screened for *S. flexneri* and *S. dysenteriae* using culture-biochemical methods, followed by species-specific PCR of culture-positive isolates (Table 2). Overall, the pooled culture-based prevalence was 50.0% (150/300; 95% CI: 44.3–55.7). Culture-based detection was highest in cloacal swabs, 69.57% (95% CI: 57.91–79.08), followed by meat, 48.60%, handlers, 48.00%, utensils, 39.13%, and wastewater, 36.67%. Among the 150 culture-positive samples, 144 were subsequently confirmed as *S. flexneri* or *S. dysenteriae* by species-specific PCR, comprising 98 *S. flexneri* and 46 *S. dysenteriae* isolates; the remaining six culture-positive isolates were negative for the species-specific PCR targets and were therefore not included in species-specific analyses. Based on the total number of original samples (n = 300), the overall sample-level prevalence of *S. flexneri* was 32.67% (98/300; 95% CI: 27.3–38.2), showing the highest detection in cloacal swabs, 46.38% (95% CI: 35.49–57.63), followed by handlers, 32.00%, meat, 30.84%, utensils, 27.54%, and wastewater, 20.00%. In contrast, the overall sample-level prevalence of *S. dysenteriae* was 15.33% (46/300; 95% CI: 11.4–19.9), peaking in cloacal swabs, 21.74% (95% CI: 13.55–32.93), and reaching the lowest level in wastewater, 3.33% (95% CI: 0.59–16.66).

**Table 2:** Prevalence of *Shigella* spp. detected by culture and PCR across different sample types.

Sample type (N)	Culture and Biochemical		PCR Positive							
	**Prevalence (%, n)**	**95% CI**	***S. flexneri***				***S. dysenteriae***			
			**Prevalence (%, n)**	**95% CI**	***χ2* -Value (df)**	***P*-value**	**Prevalence (%, n)**	**95% CI**	***χ2* -Value (df)**	***P*-value**
Cloacal swab (69)	69.57 (48)	(57.91–79.08)	46.38 (32)	(35.49–57.63)	9.07 (4)	0.059	21.74 (15)	(13.55–32.93)	5.82 (4)	0.21
Handler (25)	48.00 (12)	(29.15–67.50)	32.00 (8)	(17.19–51.59)			16.00 (4)	(6.45–34.65)		
Meat (107)	48.60 (52)	(39.35–57.94)	30.84 (33)	(22.83–40.23)			15.89 (17)	(10.16–24.07)		
Utensil (69)	39.13 (27)	(28.42–50.94)	27.54 (19)	(18.27–39.10)			13.04 (9)	(7.00–22.95)		
Waste water (30)	36.67 (11)	(21.93–54.49)	20.00 (6)	(9.54–37.27)			3.33 (1)	(0.59–16.66)		

N = total number of samples; n = number of positive samples; CI = confidence interval. Species-specific PCR was performed on culture-positive isolates (n = 150), while prevalence was calculated based on the total number of samples tested (N = 300).

#### Phenotypic detection of ESBL by DDST

Phenotypic ESBL production was assessed using the double-disk synergy test (DDST). Of the tested isolates, *S. flexneri* exhibited a characteristic keyhole-shaped extension of the inhibition zone toward the amoxicillin-clavulanic acid disk in 39 isolates (39/98; 39.8%), while *S. dysenteriae* showed the same characteristic pattern in 19 isolates (19/46; 41.3%), which were classified as phenotypic ESBL producers.

### *Prevalence of* beta-lactams*, antimicrobial resistance, efflux pump, and virulence genes in Shigella* spp.

The distribution of beta-lactams, antimicrobial resistance, efflux pump, and virulence genes varied descriptively between *S. flexneri* (n = 98) and *S. dysenteriae* (n = 46) (Figs. 2 and 3; Table 3). In *S. flexneri*, the predominant beta-lactams determinants were *bla*_TEM_ (38.78%) and *bla*_OXA_ (20.41%), followed by *MultiCase*DHA (13.27%) and *MultiCase*ACC (8.16%), whereas *bla*_CTX-M-group1_ was detected in 3.06% of isolates. In *S. dysenteriae, bla*_TEM_ (45.65%), *bla*_OXA_ (15.22%), *MultiCase*DHA (15.22%), and *MultiCase*ACC (10.87%) showed a similar distribution, with *bla*_CTX-M-group1_ detected in 4.35%. No ESBL determinant showed a significant interspecies difference after FDR adjustment (all q ≥ 0.8121). Among AMR genes, *tet(A)* and *qnrS* were highly prevalent in both species, while *sul1* and *str(A)* were more frequent in *S. dysenteriae* than *S. flexneri* (69.57% vs. 50.00%; OR = 0.44, 95% CI: 0.19–0.97; *P* = 0.0273; *q* = 0.1458 and 43.48% vs. 22.45%; OR = 0.38, 95% CI: 0.17–0.86; *P* = 0.0096; *q* = 0.1307, respectively). Similarly, *acrAB* was more prevalent in *S. dysenteriae* (80.43% vs. 60.20%; OR = 0.37, 95% CI: 0.14–0.89; *P* = 0.0163; *q* = 0.1307), whereas *tolC* showed high prevalence in both species without evidence of a difference (86.96% vs. 77.55%; OR = 0.52, 95% CI: 0.16–1.46; *P* = 0.1836; *q* = 0.6430). Virulence-associated genes were detected at low frequencies, including *ipaB* (5.10% vs. 4.35%), *ipaC* (4.08% vs. 4.35%), *ipaD* (2.04% vs. 4.35%), and *sen* (2.04% vs. 6.52%), whereas *virA* was absent in both species. No virulence-associated gene showed evidence of an interspecies difference after FDR correction (all *q* ≥ 0.8121). Overall, none of the evaluated gene-level comparisons remained statistically significant after multiple-testing correction, indicating that the observed interspecies differences were nominal and should be interpreted as exploratory associations.

**Fig. 2:**
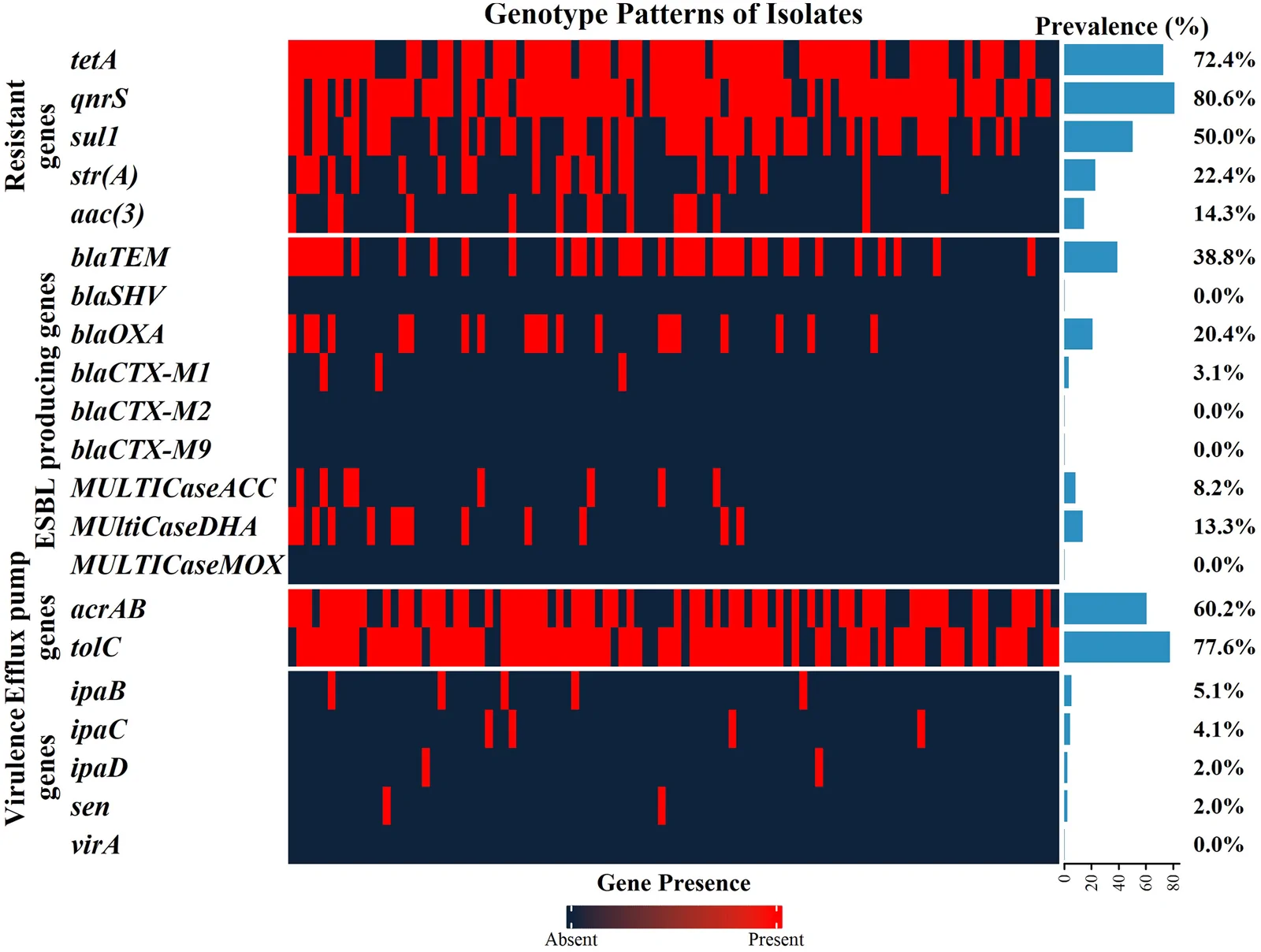
Distribution of antimicrobial resistance (AMR), ESBL-associated, efflux pump, and virulence genes among *Shigella flexneri* isolates. Integrated heatmap-bar plot showing the prevalence of AMR, ESBL, efflux pump, and virulence genes in *S. flexneri*.

**Fig. 3:**
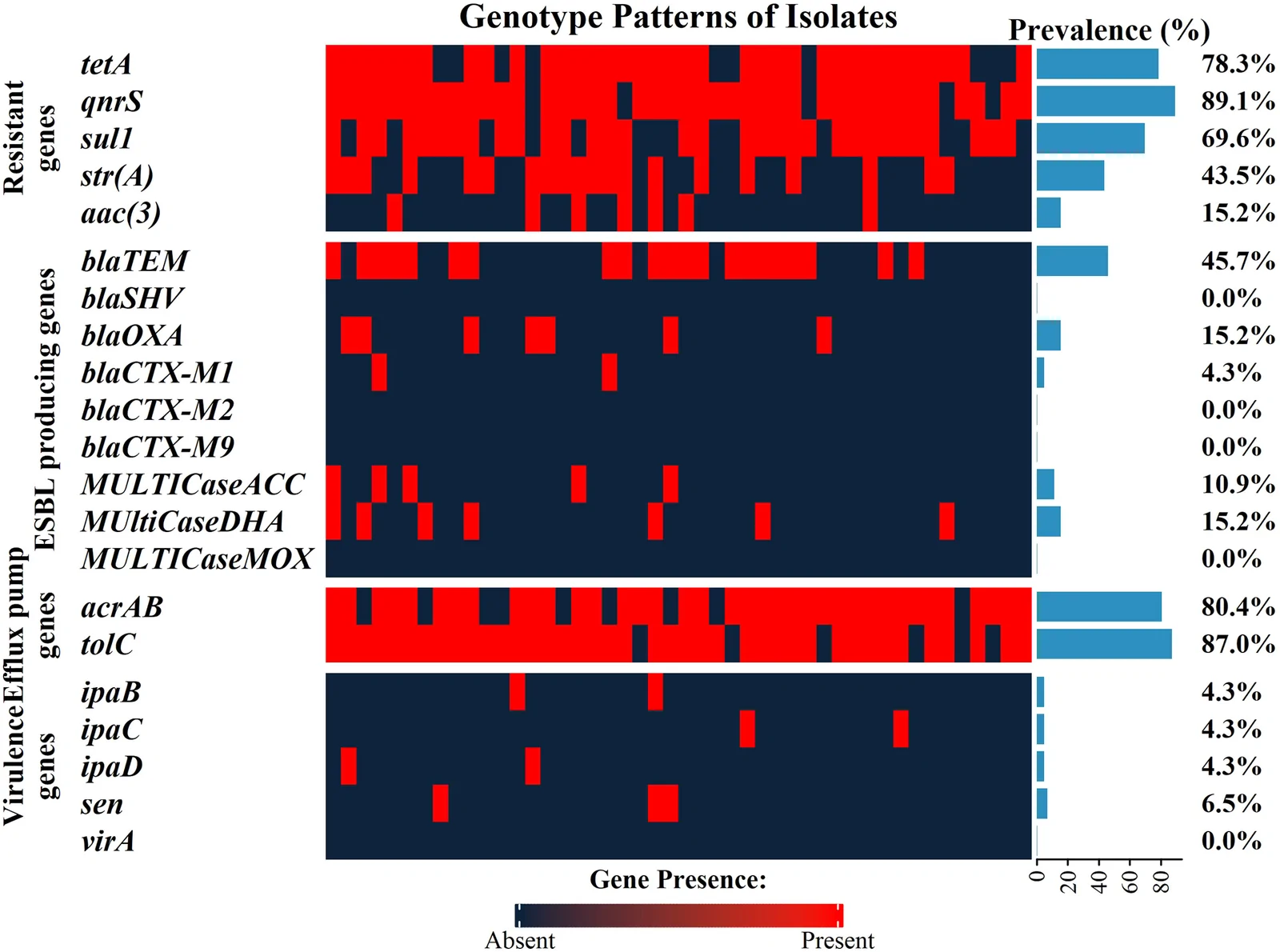
Distribution of antimicrobial resistance (AMR), ESBL-associated, efflux pump, and virulence genes among *S. dysenteriae* isolates. Integrated heatmap-bar plot showing the prevalence of AMR, ESBL, efflux pump, and virulence genes in *S. dysenteriae*.

**Table 3:** Distribution of ESBL, antimicrobial resistance, efflux pump, and virulence genes in *Shigella* isolates.

**Class**	**Resistant Genes**	***S. flexneri* (N** **=** **98) (%, n)**	***S. dysenteriae* (N** **=** **46) (%, n)**	***P*-value**	**OR (95% CI)**	**FDR q**
ESBL genes	*bla*_TEM_	38.78 (38)	45.65 (21)	0.4340	0.76 (0.35–1.63)	0.8121
	*bla*_SHV_	0.00 (0)	0.00 (0)	-	-	-
	*bla*_OXA_	20.41 (20)	15.22 (7)	0.4568	1.43 (0.52–4.34)	0.8121
	*bla*_CTX-M-group1_	3.06 (3)	4.35 (2)	0.6545ᶠ	0.70 (0.08–8.62)	0.9304
	*bla*_CTX-M-group2_	0.00 (0)	0.00 (0)	-	-	-
	*bla*_CTX-M-group9_	0.00 (0)	0.00 (0)	-	-	-
	*MultiCase*_ACC_	8.16 (8)	10.87 (5)	0.7559ᶠ	0.73 (0.20–3.02)	0.9304
	*MultiCase*_DHA_	13.27 (13)	15.22 (7)	0.7521	0.85 (0.29–2.73)	0.9304
	*MultiCase*_MOX_	0.00 (0)	0.00 (0)	-	-	-
AMR genes	*tet*(A)	72.45 (71)	78.26 (36)	0.4567	0.73 (0.28–1.77)	0.8121
	*qnrS*	80.61 (79)	89.13 (41)	0.2009	0.51 (0.14–1.55)	0.6430
	*sul1*	50.00 (49)	69.57 (32)	0.0273	0.44 (0.19–0.97)	0.1458
	*str*(A)	22.45 (22)	43.48 (20)	0.0096	0.38 (0.17–0.86)	0.1307
	*aac*(3)*-iv*	14.29 (14)	15.22 (7)	0.8826	0.93 (0.32–2.95)	1.0000
Efflux Pump genes	*acrAB*	60.20 (59)	80.43 (37)	0.0163	0.37 (0.14–0.89)	0.1307
	*tolC*	77.55 (76)	86.96 (40)	0.1836	0.52 (0.16–1.46)	0.6430
Virulence genes	*ipaB*	5.10 (5)	4.35 (2)	1.0000ᶠ	1.18 (0.18–12.87)	1.0000
	*ipaC*	4.08 (4)	4.35 (2)	1.0000ᶠ	0.94 (0.13–10.72)	1.0000
	*ipaD*	2.04 (2)	4.35 (2)	0.5927ᶠ	0.46 (0.03–6.55)	0.9304
	*sen*	2.04 (2)	6.52 (3)	0.3273ᶠ	0.30 (0.02–2.73)	0.8121
	*virA*	0.00 (0)	0.00 (0)	-	-	-

N, total number tested; n, number positive. Unmarked *P*-values were calculated using Pearson’s chi-square test; ᶠ, Fisher’s exact test (used when any expected cell count was <5); OR, odds ratio; CI, confidence interval; FDR, false discovery rate. ORs are reported with 95% CIs, with *S. flexneri* as the reference group. P-values were adjusted for multiple comparisons using the Benjamini–Hochberg FDR method. –, not applicable because the gene was absent in both species.

### Antimicrobial susceptibility profiling

The AST profiles of *S. flexneri* (n = 98) and *S. dysenteriae* (n = 46) demonstrated a consistent pattern of reduced susceptibility to commonly used first-line antibiotics (Fig. 4, Table 4). In *S. flexneri*, highest susceptibility was observed for cefixime (82.7%) and gentamicin (80.6%), followed by streptomycin (33.7%), whereas marked resistance was recorded against trimethoprim–sulfamethoxazole (92.9%), tetracycline (87.7%), azithromycin (86.7%), chloramphenicol (78.6%), levofloxacin (76.5%), and ampicillin (90% resistant). A similar pattern was observed in *S. dysenteriae*, with relatively higher susceptibility to cefixime (67.4%), gentamicin (58.7%), and streptomycin (45.7%), alongside substantial resistance to tetracycline (87.0%), trimethoprim–sulfamethoxazole (84.8%), azithromycin (76.1%), ampicillin (60.8%), and levofloxacin (56.5%), indicating limited effectiveness of several routinely used antimicrobials in both species.

**Fig. 4:**
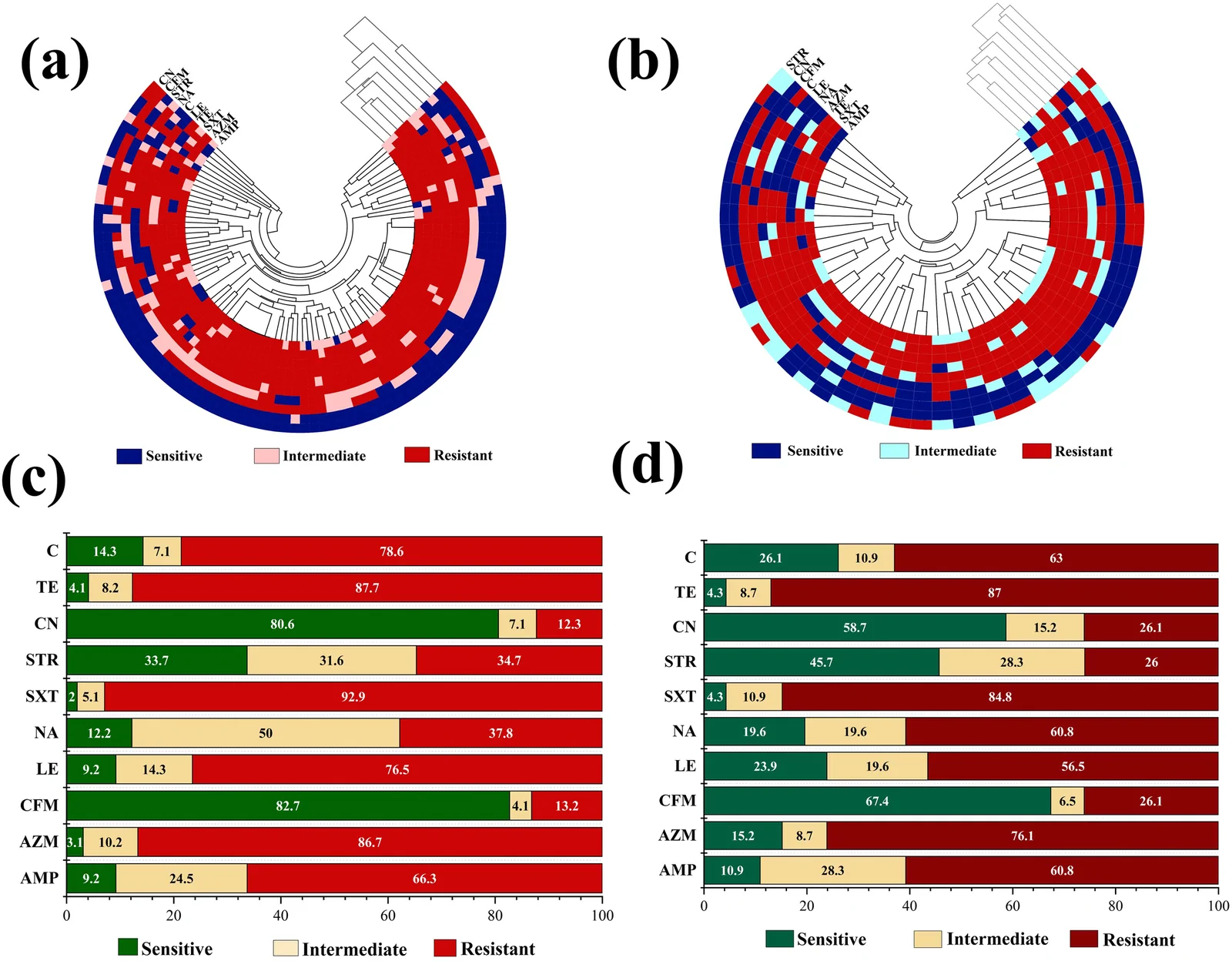
Polar heatmap and bar diagrams representing the antibiogram profiles of *S. flexneri* and *S. dysenteriae* isolates. (a) The sensitive, intermediate, and resistant patterns of *S. flexneri* isolates across different antimicrobial agents; (b) the sensitive, intermediate, and resistant patterns of *S. dysenteriae* isolates across different antimicrobial agents; (c) bar diagram showing the percentages (%) of sensitivity, resistance, and intermediate responses to different antimicrobials in *S. flexneri* isolates; (d) bar diagram showing the percentages (%) of sensitivity, resistance, and intermediate responses to different antimicrobials in *S. dysenteriae* isolates.

**Table 4:** Antimicrobial Susceptibility Patterns of *S. flexneri* and *S. dysenteriae* isolates.

**Antibiotics**	***S. flexneri* (N** **=** **98)**			***S. dysenteriae* (N** **=** **46)**		
	**S (%, n)**	**I (%, n)**	**R (%, n)**	**S (%, n)**	**I (%, n)**	**R (%, n)**
Ampicillin (AMP)	9.2 (9)	24.5 (24)	66.3 (65)	10.9 (5)	28.3 (13)	60.8 (28)
Azithromycin (AZM)	3.1 (3)	10.2 (10)	86.7 (85)	15.2 (7)	8.7 (4)	76.1 (35)
Cefixime (CFM)	82.7 (81)	4.1 (4)	13.2 (13)	67.4 (31)	6.5 (3)	26.1 (12)
Levofloxacin (LE)	9.2 (9)	14.3 (14)	76.5 (75)	23.9 (11)	19.6 (9)	56.5 (26)
Nalidixic acid (NA)	12.2 (12)	50.0 (49)	37.8 (37)	19.6 (9)	19.6 (9)	60.8 (28)
Trimethoprim-sulfamethoxazole (SXT)	2.0 (2)	5.1 (5)	92.9 (91)	4.3 (2)	10.9 (5)	84.8 (39)
Streptomycin (STR)	33.7 (33)	31.6 (31)	34.7 (34)	45.7 (21)	28.3 (13)	26.0 (12)
Gentamicin (CN)	80.6 (79)	7.1 (7)	12.3 (12)	58.7 (27)	15.2 (7)	26.1 (12)
Tetracycline (TE)	4.1 (4)	8.2 (8)	87.7 (86)	4.3 (2)	8.7 (4)	87.0 (40)
Chloramphenicol (C)	14.3 (14)	7.1 (7)	78.6 (77)	26.1 (12)	10.9 (5)	63.0 (29)

S, I, and R denote susceptible, intermediate, and resistant isolates, respectively.

### *Comparative Biofilm formation in Shigella* spp.

Biofilm formation among *S. dysenteriae* (n = 46) and *S. flexneri* (n = 98) varied across sample types and was consistently higher when assessed by Congo red agar (CRA) compared to the microtiter plate (MTP) assay. Biofilm formation is associated with reduced antimicrobial susceptibility and may contribute to the emergence of MDR, XDR or PDR strains. In *S. flexneri*, overall biofilm positivity was 37.76% (37/98) by CRA and 27.55% (27/98) by MTP, with the highest CRA detection observed in meat (54.55%) and utensils (47.37%), while MTP showed peak positivity in cloacal swabs (37.50%). In *S. dysenteriae*, biofilm formation was higher by CRA (56.52%) than MTP (36.96%), with the highest CRA positivity in meat (76.47%) and utensils (66.67%), whereas MTP showed maximum detection in cloacal swabs (53.33%) (Table 5).

**Table 5:** Biofilm formation in *Shigella* spp. from different samples.

**Sample Type**	***S. flexneri***			***S. dysenteriae***		
	**No. of isolates**	**Congo red culture (n, %)**	**Microtiter plate assay (n, %)**	**No. of isolates**	**Congo red culture (n, %)**	**Microtiter plate assay (n, %)**
Meat	33	18 (54.55)	9 (27.27)	17	13 (76.47)	5 (29.41)
Handler	8	2 (25.00)	1 (12.50)	4	1 (25.00)	1 (25.00)
Utensils	19	9 (47.37)	5 (26.32)	9	6 (66.67)	3 (33.33)
Cloacal swab	32	6 (18.75)	12 (37.50)	15	6 (40.00)	8 (53.33)
Waste water	6	2 (33.33)	0 (0.00)	1	0 (0.00)	0 (0.00)
Total	98	37 (37.76)	27 (27.55)	46	26 (56.52)	17 (36.96)

### Phenotypic characteristics of the recovered isolates and MDR, XDR, PDR, and MAR index

A total of 98 *S. flexneri* and 46 *S. dysenteriae* isolates exhibited multidrug-resistant (MDR) and possible extensively drug-resistant phenotypes, with all isolates demonstrating elevated Multiple Antibiotic Resistance Index (MARI > 0.20). In *S. flexneri*, P-XDR phenotypes were predominant, accounting for 61.22% (60/98) of isolates, while MDR phenotypes constituted 38.78% (38/98) (Table 6). The most extensive resistance profile exhibited co-resistance to nine antibiotics spanning eight antimicrobial classes (AMP-AZM-CFM-C-LE-NA-STR-SXT-TE), with a MARI value of 0.90, whereas the least extensive MDR patterns involved resistance to three antibiotics across three classes, corresponding to a MARI value of 0.30. Similarly, *S. dysenteriae* demonstrated a substantial burden of antimicrobial resistance, with MDR and P-XDR phenotypes accounting for 58.70% (27/46) and 41.30% (19/46) of isolates, respectively. The highest resistance profile likewise involved co-resistance to nine antibiotics across eight antimicrobial classes (MARI = 0.90), while the lowest resistance patterns comprised three antibiotics across three classes (MARI = 0.30).

**Table 6:** Phenotypic antibiotic resistance patterns, resistance types, and multiple antibiotic resistance index (MARI) of *S. flexneri* and *S. dysenteriae* isolates.

**Organisms**	**No. of isolates**	**%**	**Resistance type**	**Phenotypic pattern**	**Antibiotics (class)**	**MARI**
***S. flexneri***	15	15.31	P. XDR	AMP-AZM-C-LE-SXT-TE	6(6)	0.6
	9	9.18	P. XDR	AMP-AZM-C-LE-STR-SXT-TE	7(7)	0.7
	6	6.12	MDR	AZM-C-LE-SXT-TE	5(5)	0.5
	4	4.08	P. XDR	AZM-C-LE-STR-SXT-TE	6(6)	0.6
	2	2.04	MDR	AMP-AZM-C-LE-SXT	5(5)	0.5
	2	2.04	MDR	AMP-AZM-LE-SXT-TE	5(5)	0.5
	2	2.04	MDR	AZM-C-LE-NA-SXT-TE	6(5)	0.6
	2	2.04	P. XDR	AMP-AZM-C-CN-LE-NA-SXT-TE	8(7)	0.8
	2	2.04	P. XDR	AMP-AZM-C-CN-LE-STR-SXT-TE	8(7)	0.8
	2	2.04	P. XDR	AMP-AZM-C-LE-NA-SXT-TE	7(6)	0.7
	2	2.04	P. XDR	AMP-AZM-C-NA-SXT-TE	6(6)	0.6
	2	2.04	P. XDR	AMP-AZM-C-STR-SXT-TE	6(6)	0.6
	2	2.04	P. XDR	AMP-C-LE-STR-SXT-TE	6(6)	0.6
	2	2.04	P. XDR	AZM-C-LE-NA-STR-SXT-TE	7(6)	0.7
	2	2.04	P. XDR	AZM-CFM-C-LE-NA-SXT-TE	7(6)	0.7
	1	1.02	MDR	AMP-AZM-C-NA-TE	5(5)	0.5
	1	1.02	MDR	AMP-AZM-C-SXT	4(4)	0.4
	1	1.02	MDR	AMP-AZM-C-SXT-TE	5(5)	0.5
	1	1.02	MDR	AMP-AZM-LE-NA-STR-SXT	6(5)	0.6
	1	1.02	MDR	AMP-AZM-LE-NA-SXT	5(4)	0.5
	1	1.02	MDR	AMP-AZM-LE-NA-SXT-TE	6(5)	0.6
	1	1.02	MDR	AMP-AZM-LE-STR-TE	5(5)	0.5
	1	1.02	MDR	AMP-AZM-STR-SXT-TE	5(5)	0.5
	1	1.02	MDR	AMP-C-LE-NA-SXT	5(4)	0.5
	1	1.02	MDR	AMP-C-LE-SXT	4(4)	0.4
	1	1.02	MDR	AMP-C-NA-SXT-TE	5(5)	0.5
	1	1.02	MDR	AMP-CFM-C-LE-NA-SXT	6(5)	0.6
	1	1.02	MDR	AZM-C-CN-SXT-TE	5(5)	0.5
	1	1.02	MDR	AZM-C-NA-SXT-TE	5(5)	0.5
	1	1.02	MDR	AZM-CFM-C-LE-NA-SXT	6(5)	0.6
	1	1.02	MDR	AZM-CFM-CN-TE	4(4)	0.4
	1	1.02	MDR	AZM-CFM-LE-NA	4(3)	0.4
	1	1.02	MDR	AZM-CFM-LE-STR	4(4)	0.4
	1	1.02	MDR	AZM-LE-NA-SXT-TE	5(4)	0.5
	1	1.02	MDR	AZM-LE-SXT-TE	4(4)	0.4
	1	1.02	MDR	AZM-NA-SXT-TE	4(4)	0.4
	1	1.02	MDR	C-LE-NA-SXT-TE	5(4)	0.5
	1	1.02	MDR	C-LE-SXT-TE	4(4)	0.4
	1	1.02	MDR	C—NA-SXT-TE	4(4)	0.4
	1	1.02	MDR	CN-SXT-TE	3(3)	0.3
	1	1.02	MDR	LE-NA-SXT-TE	4(3)	0.4
	1	1.02	MDR	STR-SXT-TE	3(3)	0.3
	1	1.02	P. XDR	AMP-AZM-C-CN-LE-SXT-TE	7(7)	0.7
	1	1.02	P. XDR	AMP-AZM-C-CN—NA-TE	6(6)	0.6
	1	1.02	P. XDR	AMP-AZM-C-CN-SXT-TE	6(6)	0.6
	1	1.02	P. XDR	AMP-AZM-C-LE-NA-STR-SXT-TE	8(7)	0.8
	1	1.02	P. XDR	AMP-AZM-CFM-C-LE-NA-STR-SXT-TE	9(8)	0.9
	1	1.02	P. XDR	AMP-AZM-CFM-C-LE-STR-SXT-TE	8(8)	0.8
	1	1.02	P. XDR	AMP-AZM-CFM-C-NA-TE	6(6)	0.6
	1	1.02	P. XDR	AMP-AZM-CFM-C-SXT-TE	6(6)	0.6
	1	1.02	P. XDR	AMP-AZM-CN-LE-NA-SXT-TE	7(6)	0.7
	1	1.02	P. XDR	AMP-AZM-LE-NA-STR-SXT-TE	7(6)	0.7
	1	1.02	P. XDR	AMP-AZM-LE-STR-SXT-TE	6(6)	0.6
	1	1.02	P. XDR	AMP-AZM-NA-STR-SXT-TE	6(6)	0.6
	1	1.02	P. XDR	AMP-CFM-C-LE-NA-STR-SXT	7(6)	0.7
	1	1.02	P. XDR	AZM-C-NA-STR-SXT-TE	6(6)	0.6
	1	1.02	P. XDR	AZM-CFM-CN—NA-SXT-TE	6(6)	0.6
**Total**	**98**					

***S. dysenteriae***	2	4.35	MDR	AMP-C-CN-SXT-TE	5(5)	0.5
	2	4.35	MDR	AZM-C-LE-NA-SXT-TE	6(5)	0.6
	2	4.35	P. XDR	AMP-AZM-C-CN-LE-STR-SXT-TE	8(7)	0.8
	2	4.35	P. XDR	AZM-CFM-C-LE-NA-SXT-TE	7(6)	0.7
	1	2.17	MDR	AMP-AZM-C-LE-SXT	5(5)	0.5
	1	2.17	MDR	AMP-AZM-C-NA-TE	5(5)	0.5
	1	2.17	MDR	AMP-AZM-C-SXT-TE	5(5)	0.5
	1	2.17	MDR	AMP-AZM-LE-NA-STR-SXT	6(5)	0.6
	1	2.17	MDR	AMP-AZM-LE-NA-SXT	5(4)	0.5
	1	2.17	MDR	AMP-AZM-LE-NA-SXT-TE	6(5)	0.6
	1	2.17	MDR	AMP-AZM-STR-SXT-TE	5(5)	0.5
	1	2.17	MDR	AMP-C-CN	3(3)	0.3
	1	2.17	MDR	AMP-C-NA-SXT-TE	5(5)	0.5
	1	2.17	MDR	AMP-CFM-C-LE-NA-SXT	6(5)	0.6
	1	2.17	MDR	AMP-SXT-TE	3(3)	0.3
	1	2.17	MDR	AZM-C-CN-LE-SXT	5(5)	0.5
	1	2.17	MDR	AZM-CFM-C-LE-NA-SXT	6(5)	0.6
	1	2.17	MDR	AZM-CFM-CN-TE	4(4)	0.4
	1	2.17	MDR	AZM-CFM-LE-NA	4(3)	0.4
	1	2.17	MDR	AZM-CFM-LE-STR	4(4)	0.4
	1	2.17	MDR	AZM-LE-NA-SXT-TE	5(4)	0.5
	1	2.17	MDR	AZM-LE-SXT-TE	4(4)	0.4
	1	2.17	MDR	AZM-NA-SXT-TE	4(4)	0.4
	1	2.17	MDR	C-LE-NA-SXT-TE	5(4)	0.5
	1	2.17	MDR	C-SXT-TE	3(3)	0.3
	1	2.17	MDR	LE—NA-SXT-TE	4(3)	0.4
	1	2.17	MDR	STR-SXT-TE	3(3)	0.3
	1	2.17	P. XDR	AMP-AZM-C-CN-LE-NA-SXT-TE	8(7)	0.8
	1	2.17	P. XDR	AMP-AZM-C-CN—NA-TE	6(6)	0.6
	1	2.17	P. XDR	AMP-AZM-C-CN-SXT-TE	6(6)	0.6
	1	2.17	P. XDR	AMP-AZM-C-LE-NA-SXT-TE	7(6)	0.7
	1	2.17	P. XDR	AMP-AZM-C-NA-SXT-TE	6(6)	0.6
	1	2.17	P. XDR	AMP-AZM-C-STR-SXT-TE	6(6)	0.6
	1	2.17	P. XDR	AMP-AZM-CFM-C-LE-NA-STR-SXT-TE	9(8)	0.9
	1	2.17	P. XDR	AMP-AZM-CFM-C-NA-TE	6(6)	0.6
	1	2.17	P. XDR	AMP-AZM-CFM-C-SXT-TE	6(6)	0.6
	1	2.17	P. XDR	AMP-AZM-CN-LE-NA-SXT-TE	7(6)	0.7
	1	2.17	P. XDR	AMP-AZM-LE-NA-STR-SXT-TE	7(6)	0.7
	1	2.17	P. XDR	AMP-AZM-NA-STR-SXT-TE	6(6)	0.6
	1	2.17	P. XDR	AMP-CFM-C-LE-NA-STR-SXT	7(6)	0.7
	1	2.17	P. XDR	AZM-C-LE-NA-STR-SXT-TE	7(6)	0.7
	1	2.17	P. XDR	AZM-CFM-CN—NA-SXT-TE	6(6)	0.6
**Total**	46					

AMP, ampicillin; AZM, azithromycin; CFM, cefixime; C, chloramphenicol; CN, gentamicin; LE, levofloxacin; NA, nalidixic acid; STR, streptomycin; SXT, trimethoprim-sulfamethoxazole; and TE, tetracycline.

### Pairwise associations among genotypic and phenotypic traits in S. dysenteriae and S. flexneri

Pairwise associations among 26 variable traits (16 genes and 10 antimicrobial resistance phenotypes; five invariant genes excluded) were assessed separately in *S. dysenteriae* (n = 46) (Fig. 5) and *S. flexneri* (n = 98) (Fig. 6) using the phi coefficient and Fisher’s exact test, with Benjamini–Hochberg false discovery rate (FDR) correction across 325 comparisons per species. Nominally significant associations (*P* < 0.05) were detected for 12 pairs (3.7%) in *S. dysenteriae* and 23 pairs (7.1%) in *S. flexneri*; however, none remained significant after FDR correction (all *q* ≥ 0.05 and *q* ≥ 0.176, respectively). The strongest species-specific associations were observed for *bla*_OXA_–*ipaD* (φ = 0.50), *acrAB*–AMP (φ = 0.39), and LE–NA (φ = 0.38) in *S. dysenteriae*, and *bla*_OXA_–*bla*_DHA_ (φ = 0.40, *q* = 0.18), *bla*_TEM_–*sul1* (φ = 0.34), and CFM–SXT (φ = −0.36) in *S. flexneri*. Two pairs, *tet*(A)–*str*(A) (φ = 0.36 and 0.28) and *str*(A)–*tolC* (φ = 0.34 and 0.29), showed nominal significance in both species, suggesting potentially shared patterns of co-occurrence. In contrast, canonical gene–phenotype pairs showed negligible or weak associations in both species, including *tet*(A)–TE (φ = −0.02 and −0.09), *sul1*–SXT (φ = 0.11 and −0.04), *bla*_TEM_–AMP (φ = 0.02 and −0.01), and *qnrS*–NA (φ = 0.15 and 0.12). Overall, these associations should be interpreted as exploratory and hypothesis-generating, given the relatively small species-specific sample sizes, multiple comparisons, and high prevalence of several traits.

**Fig. 5:**
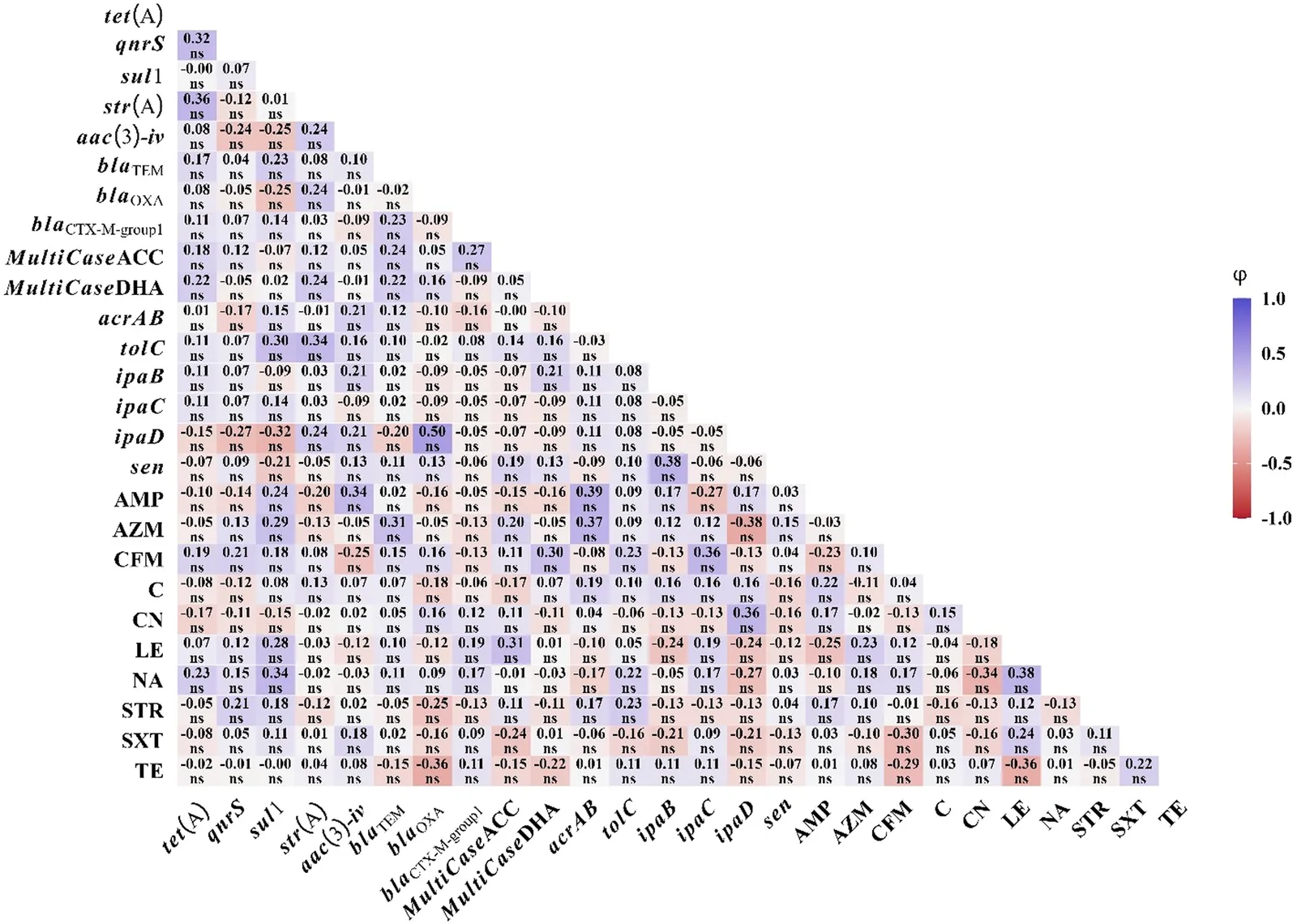
Pairwise associations among resistance genes, virulence genes, and antimicrobial resistance phenotypes in *S. dysenteriae* (n = 46). Tile colour indicates the phi coefficient (φ; blue, positive; red, negative), with values showing φ and BH-FDR significance (*q* < 0.05 *, < 0.01 **, < 0.001 ***; ns, not significant). Associations were assessed using Fisher’s exact test; invariant variables were excluded.

**Fig. 6:**
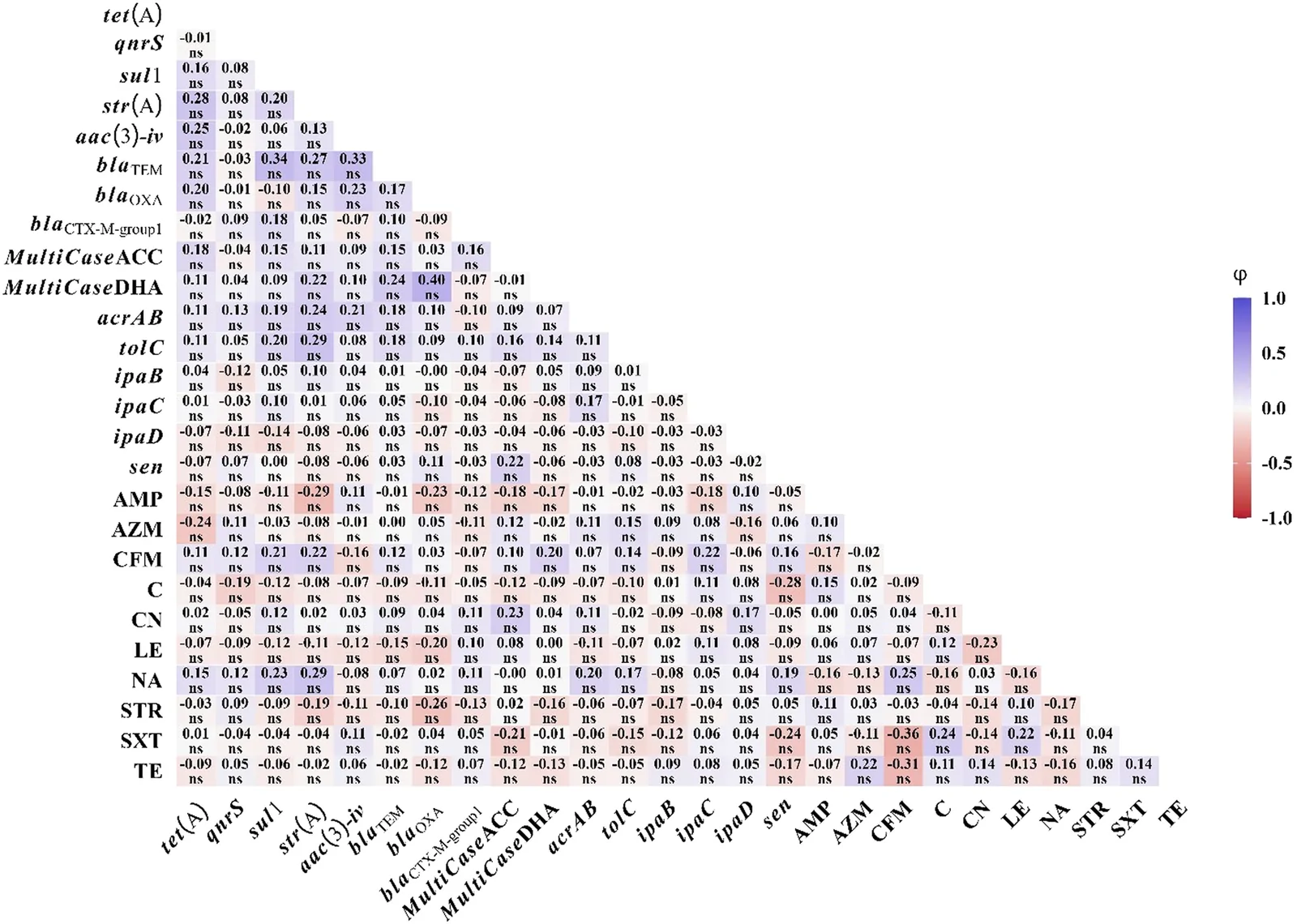
Pairwise associations among resistance genes, virulence genes, and antimicrobial resistance phenotypes in *S. flexneri* (n = 98). Tile colour indicates the phi coefficient (φ; blue, positive; red, negative). Values show φ with BH-FDR significance (*q* < 0.05, *, < 0.01, **, < 0.001, ***; ns, not significant). Associations were assessed using Fisher’s exact test; invariant variables were excluded.

## Discussion

*S. flexneri* and *S. dysenteriae* are important human-adapted enteric pathogens responsible for bacillary dysentery, with *S. flexneri* being the most commonly isolated species in endemic regions and *S. dysenteriae* often associated with more severe clinical outcomes due to its potent Shiga toxin production (Bengtsson et al., 2022; Pakbin et al., 2023; Hendrick et al., 2025). The detection of these species in food-associated environments highlights potential contamination risks and underscores the importance of monitoring antimicrobial-resistant strains at the retail level to support public health safety.

The present study demonstrates a high and widespread burden of *S. flexneri* and *S. dysenteriae* across multiple sampling matrices within the investigated retail poultry-handling environment (cloacal swabs, meat, handlers, utensils, and wastewater), indicating widespread occurrence across food-contact, occupational, and environmental matrices. The overall culture-based *Shigella* prevalence of 50.0% reflects substantial contamination within slaughter and processing settings, with the highest detection in cloacal swabs (69.57%), suggesting that poultry intestinal contents may represent an important potential contributor to contamination, although source attribution cannot be established from the present cross-sectional design. Substantial detection in meat (48.60%), handlers (48.00%), utensils (39.13%), and wastewater (36.67%) suggests the potential for local cross-contamination during slaughtering, meat handling, and waste management, where inadequate hygiene practices facilitate bacterial transfer between biological and non-biological surfaces. Meat contamination may occur during evisceration or contact with contaminated surfaces, while repeated handling and utensil reuse may facilitate transfer and persistence; wastewater detection may reflect the accumulation and discharge of contaminated organic material. These mechanisms are plausible but were not directly evaluated in this study. This prevalence substantially exceeds the 18.75% reported in wild waterfowl and migratory birds in Bangladesh (Karmoker et al., 2023). Such differences may reflect variation in host ecology, sampling context, and environmental exposure rather than directly demonstrated differences in transmission pressure. Molecular analysis identified *S. flexneri* as the predominant species, suggesting potential contamination of occupational and food-contact environments within the retail setting. Its predominance may reflect species- or strain-level differences in ecological persistence or occurrence within the sampled environment, although these mechanisms were not assessed. In contrast, *S. dysenteriae* showed lower overall prevalence (15.33%), peaking in cloacal swabs (21.74%) and declining markedly in wastewater (3.33%). Within the sampled retail poultry-handling environment, *S. flexneri* was more frequently detected than *S. dysenteriae*, which was also identified across multiple investigated matrices, including environmental samples. These findings demonstrate species-specific differences in occurrence within the study setting but do not establish differences in their source, transmission potential, or epidemiological importance. In Bangladesh, nationwide surveillance (2014–2022) similarly reported *S. flexneri* as predominant (79.5%), whereas *S. dysenteriae* accounted for only 0.5% of clinical cases (Nyarkoh et al., 2024). Conversely, a hospital-based study found higher *S. dysenteriae* type 1 (59%) than *S. flexneri* (39%) among bloody diarrhoea patients (Ud-Din et al., 2013). The observed variation is likely attributable to heterogeneity in study populations, ecological reservoirs, and transmission dynamics across clinical, environmental, and animal-associated settings, collectively shaping species-specific distribution patterns.

Our investigation revealed high levels of resistance to several commonly used antimicrobial agents in poultry-associated *Shigella*, with both *S. flexneri* and *S. dysenteriae* exhibiting near-universal resistance to historically effective agents. Both species exhibited critical resistance to trimethoprim–sulfamethoxazole, tetracycline, azithromycin, and levofloxacin. These findings align with reports on avian reservoirs in Bangladesh, which show high resistance to tetracycline (86.67%), azithromycin (80%), ampicillin (66.67%), and ciprofloxacin (40%) (Karmoker et al., 2023). The broadly comparable resistance profiles across avian-associated settings suggest that similar resistance patterns may occur in different ecological contexts (Karmoker et al., 2023). These findings demonstrate substantial resistance to several commonly used antimicrobial agents; however, antimicrobial exposure was not assessed, precluding direct attribution of these resistance patterns to antimicrobial selection pressure or overuse/misuse.

The present study demonstrates a heterogeneous distribution of ESBL, antimicrobial resistance (AMR), efflux pump, and virulence-associated genes between *S. flexneri* and *S. dysenteriae*, reflecting both shared resistance backbones and species-specific adaptive trends. In *S. flexneri*, the prevalence of *bla*_TEM_ (38.78%) was higher than *bla*_OXA_ (20.41%), while *bla*_CTX-M-group1_ (3.06%) was detected at a low level, whereas, similarly in *S. dysenteriae, bla*_TEM_ (45.65%) was the most frequent gene followed by *bla*_OXA_ (15.22%). These findings are consistent with studies that report *bla*_TEM_ and *bla*_OXA_ as the predominant ESBL types in *Shigella* (Somda et al., 2025). Regionally, studies from Africa reported *bla*_TEM_ (25.9%) and *bla*_OXA-1_ (25.7%) as the most common ESBL determinants, with a comparatively lower prevalence of *bla*_CTX-M_ (10.8%) (Somda et al., 2025). Similarly, in Jinan, China, exceptionally high frequencies of *bla*_TEM-1_ (67.6%) and *bla*_OXA-30_ (100%) have been documented among ESBL-producing *S. flexneri* isolates (Bian et al., 2019). In Wuhan, China, *bla*_OXA_ (50%) and *bla*_TEM_ (40%) were also reported as the dominant resistance genes among ampicillin-resistant isolates (Deng et al., 2025). The widespread distribution of *bla*_TEM_ and *bla*_OXA_ highlights their stable evolutionary persistence in *Shigella* spp., whereas the lower occurrence of *bla*_CTX-M_ variants suggests ongoing but heterogeneous dissemination across regions. Among AMR genes, *tetA* was highly prevalent in both *S. flexneri* (72.45%) and *S. dysenteriae* (78.26%), indicating a high prevalence of the *tetA* resistance determinant in both species, with slightly higher carriage in *S. dysenteriae*. Similarly, *qnrS* showed very high distribution in *S. dysenteriae* (89.13%) compared to *S. flexneri* (80.61%), suggesting a marginally greater fluoroquinolone resistance potential in this species. In contrast, *sul1* (69.57% vs. 50.00%) and *strA* (43.48% vs. 22.45%) were markedly more prevalent in *S. dysenteriae*, indicating enhanced accumulation of sulfonamide- and streptomycin-resistance determinants relative to *S. flexneri*. These findings partially align with previous reports, although notable geographic and ecological variability exists. In Bangladesh, *tet*A (69.23%) has been reported in MDR *Shigella* from avian reservoirs, slightly lower than the present detection in *S. dysenteriae* (Karmoker et al., 2023). Conversely, markedly lower prevalence has been reported in other regions, where *qnr*S and *qnr*A were detected in 37.5% and 56.3% of *Shigella* isolates in Iraq (Al-Khafaji et al., 2024), while in Iran, *qnr*A and *qnr*S were each identified in 41% of pediatric *Shigella* isolates (Gonabadi et al., 2024). A global meta-analysis reported higher prevalence of *str*A-*str*B in *S. dysenteriae* (12.9%) than *S. flexneri* (6.4%) (Ayele et al., 2025), along with *sul2* (14.5% vs. 3.9%), respectively. In Kolkata, India, *qnrS* was detected in 62% of *Shigella* isolates from environmental water (Roy et al., 2020). Overall, the differential distribution of AMR genes between the two species indicates species-level differences in resistance-gene carriage; however, these differences cannot be directly attributed to antimicrobial selection pressure or greater epidemiological resilience in the absence of exposure and longitudinal data. Efflux pump genes were widely distributed in both *Shigella* species, with *acr*AB showing higher prevalence in *S. dysenteriae* (80.43%) than *S. flexneri* (60.20%), indicating stronger efflux-mediated multidrug resistance (MDR) potential in *S. dysenteriae. tolC* remained highly prevalent in both *S. dysenteriae* (86.96%) and *S. flexneri* (77.55%), consistent with reports from China (Yang et al., 2008), supporting its key role as the outer membrane channel of the *Acr*AB–*Tol*C efflux system in broad-spectrum resistance. In contrast, the low and comparable prevalence of virulence genes (*ipa*B, *ipa*C, *ipa*D, *sen*; 2.04%–6.52%) in both species suggests limited detection of the investigated virulence markers, rather than reduced overall pathogenic potential. Pairwise analysis identified several nominal associations among resistance genes, efflux-related determinants, virulence markers, and resistance phenotypes, but none remained significant after Benjamini–Hochberg FDR correction. The *tet*(A)–*str*(A) and *str*(A)–*tolC* pairs showed nominal associations in both species, suggesting potentially shared patterns of trait co-occurrence. However, these findings should be interpreted cautiously because of the relatively small species-specific sample sizes, multiple comparisons, and high prevalence of several traits. Weak or negligible associations were observed for several canonical gene–phenotype pairs, and the observed patterns should therefore be considered exploratory rather than evidence of confirmed genetic linkage or mechanism.

The present study demonstrated notable biofilm-forming ability in both species, with higher prevalence in *S. dysenteriae* than in *S. flexneri* indicating stronger biofilm-forming potential in *S. dysenteriae*. Across sample types, meat and utensils showed consistently higher CRA-based detection, while MTP positivity peaked in cloacal swabs, reflecting methodological and ecological variability. The higher CRA positivity in meat and utensils may be compatible with repeated exposure to contaminated material and surface persistence, whereas the higher MTP positivity in cloacal samples may reflect differences in bacterial load or physiological conditions. These findings align with reports from Gondar, Ethiopia, where *Shigella*-contaminated meat and swab samples showed 27.8% MDR isolates (Garedew et al., 2016), and with studies from Iran demonstrating biofilm formation in *S. flexneri* clinical isolates (Jalili et al., 2019), supporting the link between biofilm production and antimicrobial resistance.

A substantial burden of antimicrobial resistance was observed in both *S. flexneri* and *S. dysenteriae*, with all isolates showing MARI values >0.20. The MDR prevalence in *S. flexneri* and *S. dysenteriae* falls within the range reported in previous regional studies, being lower than that documented in Bangladesh (87.0%) (Afrad et al., 2024) but broadly comparable to East Africa (67.2%) (Ayele et al., 2023) and Asia (59.8%) (Salleh et al., 2022), indicating moderate-to-high endemic resistance in similar settings. In contrast, the prevalence of possible XDR phenotypes was markedly higher in the present study, particularly in *S. flexneri* (61.22%), compared to reports from the USA (17%) (Lu et al., 2026), suggesting a substantially greater accumulation of extensively resistant phenotypes in this study population. This disparity may be attributed to variations in antimicrobial usage patterns, infection prevention and control practices, environmental contamination, and the dynamics of horizontal gene transfer in different ecological and healthcare settings. The high proportions of MDR and possible XDR isolates indicate extensive co-resistance across multiple antimicrobial classes. Differences from previous reports may reflect variation in antimicrobial exposure, study populations, sampling matrices, resistance definitions, and local ecological conditions, although these factors were not directly assessed in the present study.

## Limitations

This study has several limitations. The restricted geographic setting may limit the generalizability of the findings, while feasible sampling and unequal sample allocation across matrices may have introduced selection bias and limited direct comparisons between sample types. Multiple samples may have originated from the same retail shops, but the absence of shop-level identifiers precluded adjustment for potential within-shop clustering. The lack of farm-, supplier-, or batch-level traceability prevented identification of upstream contamination sources, while the absence of direct antimicrobial-use and hygiene data limited inference regarding the drivers of resistance and contamination. The cross-sectional design further precludes assessment of temporal trends, causality, or transmission direction. Finally, culture- and PCR-based methods may be influenced by bacterial recovery, viability, DNA quality, and assay target selection; therefore, detection should be interpreted as evidence of occurrence rather than definitive evidence of transmission or epidemiological linkage.

## Conclusion

This study demonstrates the occurrence of *S. flexneri* and *S. dysenteriae* across multiple matrices within the retail poultry-handling environment, with their distribution consistent with potential local contamination and bacterial transfer. However, the cross-sectional design and absence of source or production-chain linkage preclude inference of transmission direction or source. Both species exhibited substantial multidrug and possible extensively drug-resistant phenotypes, accompanied by elevated MARI values indicating frequent resistance to multiple antimicrobial agents; however, antimicrobial selection pressure could not be established without direct exposure data. Molecular analysis revealed diverse ESBL and resistance determinants, with *S. dysenteriae* showing a comparatively higher accumulation of AMR determinants and biofilm-forming potential. Pairwise associations were exploratory, and none remained significant after Benjamini–Hochberg FDR correction, indicating no robust evidence of association among the tested traits after accounting for multiple comparisons. Collectively, these findings underscore the public health relevance of retail-associated *Shigella* and support strengthened hygiene and sanitation, responsible antimicrobial use, and integrated One Health surveillance at the retail level.

## Funding

This study did not receive any funding.

## Ethical statement

Animal Experimentation and Ethics Committee (AEEC) at Sylhet Agricultural University, Bangladesh, approved both the study protocol and the poultry meat examinations with an approval number #AUP2025006.

## Disclosures

The authors declare that they have no known competing financial interests or personal relationships that could have appeared to influence the work reported in this paper.

## Authors’ contributions

**Suchona Akter** carried out the experiments, processed and analyzed the data, prepared the figures, and wrote the initial draft of the manuscript. **Md. Hasan Ali** performed software-based analyses, compiled and organized the data and wrote the initial draft of the manuscript. **Hemayet Hossain** processed the data, prepared the figures and compiled and organized the data, revised and refined the manuscript. **Md. Shahidur Rahman Chowdhury** assisted with part of the experimental work, compiled and organized the data, and revised and refined the manuscript. **Mohammad Nahian Rahman** assisted with part of the experimental work, processed and analyzed the data, and wrote the initial draft of the manuscript. **Sumaya Shargin Khan** assisted with part of the experimental work, processed and analyzed the data, and wrote the initial draft of the manuscript. **Afroza Sultana Nitu** assisted with part of the experimental work, processed and analyzed the data, and wrote the initial draft of the manuscript. **Tanvir Ahmad** assisted with part of the experimental work, processed and analyzed the data, and wrote the initial draft of the manuscript. **Md. Mukter Hossain** provided technical support for the methods, supervised the study, wrote the initial draft of the manuscript and revised and refined the manuscript. **Md Bashir Uddin** provided technical support for the methods, supervised the study, wrote the initial draft of the manuscript and revised and refined the manuscript. **Md. Mahfujur Rahman** as the corresponding author, designed and supervised the study, coordinated the research project, obtained financial support, confirmed the results, compiled and organized the data and revised and refined the manuscript. All authors have reviewed and approved the final submitted manuscript. All authors are accountable for all aspects of the work and will ensure that questions related to the accuracy or integrity of any part of the article are appropriately investigated and resolved.

## Footnotes

Supplementary material associated with this article can be found, in the online version, at doi:10.1016/j.psj.2026.107935.
