# Supplementary material for: Occurrence and characterization of biofilm-forming and multidrug-resistant Shigella spp. in retail poultry meat and associated environments in Sylhet, Bangladesh
Source: Poult Sci. 2026 Oct 9;105(12):107935. doi: 10.1016/j.psj.2026.107935 (PMC13725472; doi:10.1016/j.psj.2026.107935)
Supplement: Supplementary file 1 [file mmc1.docx]

**Supplementary Table 1**: Biochemical test findings and interpretations.

| **Biochemical test** | **Biochemical test result for *Shigella* spp.** | **Interpretation for presumptive *Shigella* identification** |
| --- | --- | --- |
| Methyl Red (MR) | Positive (+) | Supports *Shigella* identification |
| Motility | Negative (−) | Supports *Shigella* identification |
| Citrate utilization | Negative (−) | Supports *Shigella* identification |
| Triple Sugar Iron (TSI) | K/A (alkaline slant/acid butt) | Consistent with *Shigella* |
| H₂S production | Negative (−) | Supports *Shigella* identification |
| Gas production | Negative (−) | Supports *Shigella* identification |
| Indole | Variable (+/−) | Not used alone for confirmation |
| Voges–Proskauer (VP) | Negative (−) | Supports *Shigella* identification |

**Supplementary Table 2.** Break point and disc potency of the selected antibiotics of this study based on CLSI 2023 for *Shigella* spp*.*

| **Antimicrobial class** | **Antibiotics** | **Disc potency (ug)** | **Interpretation** | | |
| --- | --- | --- | --- | --- | --- |
|  |  |  | **Sensitive (mm)** | **Intermediate**  **(mm)** | **Resistant**  **(mm)** |
| Penicillin | Ampicillin (AMP) | 10 | ≥ 17 | 14-16 | ≤13 |
| Cephalosporin | Cefixime (CFM) | 30 | ≥ 19 | 16-18 | ≤ 15 |
| Aminoglycosides | Gentamicin (CN) | 10 | ≥ 18 | 15–17 | ≤ 14 |
|  | Streptomycin (STR) | 10 | ≥15 | 12-14 | ≤ 11 |
| Tetracyclines | Tetracycline (TE) | 30 | ≥15 | 12-14 | ≤ 11 |
| Macrolides | Azithromycin (AZM) | 15 | ≥ 16 | 11-15 | ≤ 10 |
| Fluroquinolones | Levofloxacin (LE) | 5 | ≥21 | 17-20 | ≤ 16 |
| Quinolones | Nalidixic acid (NA) | 30 | ≥19 | 14-18 | ≤ 13 |
| Folate pathway antagonists | Trimethoprim- Sulfamethoxazole (SXT) | 1.25/23.75 | ≥16 | 11-15 | ≤ 10 |
| Phenicols | Chloramphenicol (C) | 30 | ≥18 | 13-17 | ≤ 12 |

**Supplementary Fig. 1.** Morphology of *Shigella* spp. on SS agar(A), XLD agar (B), Nutrient Agar (C); (D) MIU test, (E) VP test, (F) Citrate utilization test, (G) MR test.

**Supplementary Fig. 2.** Molecular detection of *Shigella* spp. from multiplex PCR test. M and N stand for DNA marker and negative control respectively

**Supplementary Fig. 3.** Kirby-Bauer test to assess the zone of inhibition for different antibiotics against *Shigella* spp.

**Supplementary Fig. 4.** Amplified DNA of ESBL genes of *Shigella* spp. from multiplex PCR. M and N stand for DNA marker and negative control respectively.

**Supplementary Fig. 5.** Amplified DNA of antibiotic-resistant genes of *Shigella* spp. from 5 uniplex PCR. M and N stand for DNA marker and negative control respectively.

**Supplementary Fig. 6.** Amplified DNA of Efflux pump genes of *Shigella* spp. from 2 uniplex PCR. M and N stand for DNA marker and negative control respectively.

**Supplementary Fig. 7.** Biofilm (A) and Non-biofilm (B) formation by Congo-red culture. (C) Microtitre plate assays for detection of biofilm formation.
